# Impact of the SARS-CoV-2 pandemic and associated restrictions on Pediatric Emergency Department utilization in Sardinia: a retrospective bicentric observational study

**DOI:** 10.1186/s13052-022-01225-6

**Published:** 2022-03-03

**Authors:** Roberto Antonucci, Maria Grazia Clemente, Luca Antonucci, Alessandro Canetto, Stefania Mastromattei, Noemi Chiapello, Nadia Vacca, Laura Saderi, Giovanni Sotgiu, Cristian Locci

**Affiliations:** 1 Pediatric Clinic, Department of Medical, Surgical and Experimental Sciences, University of Sassari, Sassari, Italy; 2 Pediatric Emergency Medicine, Emergency Department, ARNAS G. Brotzu, Cagliari, Italy; 3 Academic Department of Pediatrics, Children’s Hospital Bambino Gesu, University of Rome “Tor Vergata”, Rome, Italy; 4 Clinical Epidemiology and Medical Statistics Unit, Department of Medical, Surgical and Experimental Sciences, University of Sassari, Sassari, Italy

**Keywords:** SARS-CoV-2, COVID-19, Pandemic, Children, Emergency Department, Visits

## Abstract

**Background:**

The COVID-19 pandemic and associated public health measures have had a profound impact on health systems worldwide. The aim of this study was to assess quantitative and qualitative changes in Pediatric Emergency Department (PED) visits in Sardinia, Italy, during the early period of the COVID-19 pandemic.

**Methods:**

We retrospectively investigated the number and characteristics of visits to two major Sardinian PEDs, in the periods January-June 2020 and January-June 2019.

**Results:**

From January to June 2020, 8399 PED visits with 1160 hospital admissions (13.8% of PED visits) were registered, compared with 15,692 PED visits (Δ = -46.5%) and 1819 hospital admissions (11.6% of PED visits) occurring from January to June 2019.

Comparing January-June 2020 with January-June 2019, we found differences in the percentage of visits for age groups, and significant changes in the proportion of triage codes, with a decrease in green codes (72.1% *vs* 74.2%, respectively) and an increase in white codes (19.0% *vs* 16.5%, respectively). Moreover, in the period January-June 2020, the frequency of skin disorders and acute respiratory disease significantly decreased, while the frequency of trauma, acute surgical disease, intoxication, and neuropsychiatric disease significantly increased.

**Conclusions:**

After the beginning of the Italian lockdown, we observed a marked drop in the number of PED visits, an increase in hospital admission rate, and radical changes in the reason for visit.

## Introduction

The emergence of the Severe Acute Respiratory Syndrome Coronavirus 2 (SARS-CoV-2), and the resulting COVID-19 pandemic, has led to the adoption of extreme containment measures in several countries worldwide, which have changed daily activities and healthcare delivery.

Dong et al. [1] reported that children can be infected by SARS-CoV-2, but clinical manifestations are usually less severe than those occurring in adults, as confirmed by Italian findings [2]. This could explain the low rate of COVID-19-related emergency department (ED) visits in children and adolescents.

The number of ED visits has decreased after the beginning of the pandemic worldwide [3, 4]. In the USA, the total number of non-COVID-19 ED visits fell by 42% for adults during the early pandemic period when compared with the corresponding period of the previous year [3]; however, pediatric ED visits declined even more markedly [5].

Italy was the first European country to be hit by the pandemic. Italian government implemented strict lockdown measures from 9 March to 3 May 2020 [6], when the absolute number of pediatric ED visits for both urgent and non-urgent cases decreased [4, 7], and the rates of urgent triage codes and daily hospital admissions increased [7]. Multiple factors have been suggested as potential contributors to the sharp decline in pediatric ED visits, including lower incidence of communicable diseases and fewer injuries resulting from social distancing, reduced activity and travel, patients' fear of contracting COVID-19 in the ED, and the transition to telehealth as a substitute for face-to-face healthcare [8]. Moreover, data from pediatric providers have shown that approximately 1 in 3 presentations for emergency medical care was perceived as delayed [9].

So far, there are no published data regarding the impact of the COVID-19 pandemic and associated restrictions on pediatric ED utilization in the Mediterranean island of Sardinia.

The aim of the present study was to assess in detail quantitative and qualitative changes in pediatric ED visits in Sardinia, during the early period of the COVID-19 pandemic.

## Methods

A retrospective observational study was conducted to assess visits of patients aged less than 16 years at the two major pediatric EDs in Sardinia, Italy, during the periods January-June 2020 and January-June 2019. In particular, we included data from the pediatric ED at the University Hospital in Sassari and the pediatric ED at the Brotzu Hospital in Cagliari, which are located in the northern and southern parts of Sardinia island, respectively. Data per visit and not for unique patients were collected.

Demographic, epidemiological, and clinical data were anonymously abstracted using the software Areas® (Engineering Ingegneria Informatica, Rome, Italy). ED visits were stratified by age, sex, triage code, outcome (discharge after ED visit *vs* hospital admission), and main reason for visit. Four age groups were considered: < 2 years, 2–5 years, 6–11 years, and > 11 years. The national four-level category triage system was adopted: white (not urgent), green (minor urgency), yellow (urgent), and red (emergency). Reasons for ED visits were grouped into the following 14 categories: acute respiratory disease, gastrointestinal disease, trauma, skin disorder, neuropsychiatric disease, fever, pain, cardiac disease, acute surgical disease, intoxication, ocular disease, endocrine and metabolic disorder, osteoarticular disease, miscellaneous.

The data pertaining to ED visits during the first semester of 2020 were compared with those of the corresponding semester of 2019. Subsequently, the first semester of 2020 was divided into three two-month periods: January–February (pre-lockdown period), March–April (lockdown period), and May–June (immediate post-lockdown period); these two-month periods were compared with the corresponding periods in 2019.

### Definitions

Hospital admissions: absolute number of hospital admissions.

Hospital admission rate: number of hospital admissions per 100 ED visits.

### Statistical analysis

Demographic, epidemiological, and clinical characteristics were described with absolute and relative (percentages) frequencies. Qualitative variables were compared using the chi-square test. A two-tailed p-value < 0.05 was considered statistically significant. All statistical analyses were carried out using the STATA software version 17 (StataCorp LLC, TX).

## Results

**Fig. 1:**
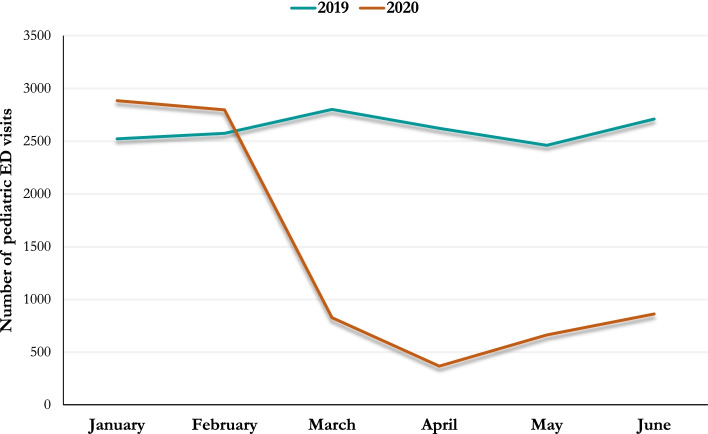
Temporal trends of number of visits to pediatric ED in the periods January-June 2020 and January-June 2019

The total number of ED visits fell from 15,692 during the first semester of 2019 to 8,399 during the same semester of the year 2020 (-46.5%) (Table 1). The monthly number of ED visits dropped from > 2,500 during the pre-lockdown period to < 1,000 during both the lockdown and the immediate post-lockdown period (Fig. 1).

**Table 1:** Descriptive analysis of demographic and clinical data: 1^st^ semester 2020 compared to 1^st^ semester 2019

*n* = *24,091*	2019	2020	*p*-value	Δ
***1° semester***				
* N. visits to PED, n (%)*	15,692 (65.1)	8399 (34.9)	-	-46.5%
* Males, n (%)*	8484 (54.1)	4481 (53.4)	0.30	-47.2%
* Age* < *2 years, n (%)*	4518 (28.8)	2804 (33.4)	< 0.0001	-37.9%
* Age 2–5 years, n (%)*	5983 (38.1)	2747 (32.7)	< 0.0001	-54.1%
* Age 6–11 years, n (%)*	4033 (25.7)	2263 (26.9)	0.04	-44.9%
* Age* > *11, n (%)*	1158 (7.4)	585 (7.0)	0.25	-49.5%
**Triage code**				
* Red, n (%)*	36 (0.2)	20 (0.2)	1.00	-44.4%
* Yellow, n (%)*	1424 (9.1)	727 (8.7)	0.30	-49.0%
* Green, n (%)*	11,646 (74.2)	6059 (72.1)	0.0004	-48.0%
* White, n (%)*	2586 (16.5)	1593 (19.0)	< 0.0001	-38.4%
* Discharge home, n (%)*	13,873 (88.4)	7239 (86.2)	< 0.0001	-47.8%
* Hospital admission, n (%)*	1819 (11.6)	1160 (13.8)	< 0.0001	-36.2%
* Death, n (%)*	0 (0.0)	0 (0.0)	-	-
**Reason for PED visit**				
* Acute respiratory disease*	6448 (41.1)	3058 (36.4)	< 0.0001	-52.6%
* Gastrointestinal disease*	2598 (16.6)	1336 (15.9)	0.16	-48.6%
* Trauma*	1629 (10.4)	1177 (14.0)	< 0.0001	-27.8%
* Skin disorders*	1526 (9.7)	575 (6.8)	< 0.0001	-62.3%
* Miscellaneous*	621 (4.0)	463 (5.5)	< 0.0001	-25.4%
* Neuropsychiatric disease*	650 (4.1)	406 (4.8)	0.01	-37.5%
* Fever*	412 (2.6)	224 (2.7)	0.64	-45.6%
* Ocular disease*	363 (2.3)	221 (2.6)	0.15	-39.1%
* Endocrine and metabolic disorder*	317 (2.0)	172 (2.1)	0.60	-45.7%
* Pain*	286 (1.8)	191 (2.3)	0.008	-33.2%
* Cardiac disease*	295 (1.9)	153 (1.8)	0.59	-48.1%
* Acute surgical disease*	218 (1.4)	185 (2.2)	< 0.0001	-15.1%
* Osteoarticular disease*	251 (1.6)	145 (1.7)	0.56	-42.2%
* Intoxication*	78 (0.5)	93 (1.1)	< 0.0001	19.2%

*PED* Pediatric Emergency Department

The data pertaining to the first, second and third bimesters of 2020, and those of the corresponding periods of 2019, are shown in Tables 2, 3 and 4. The number of visits was slightly higher in January–February 2020 (+ 11.5%). Conversely, a marked drop was observed in March–April 2020 when compared to both the pre-lockdown period (January–February 2020) (-79.0%) (Table 5) and March–April 2019 (-78.0%) (Table 3). During the immediate post-lockdown, the number of visits was lower than that observed both during the pre-lockdown period (-73.2%) and during May–June 2019 (-70.5%) (Table 4, Fig. 1).

**Table 2:** Descriptive analysis of demographic and clinical data: 1^st^ bimester 2020 compared to 1^st^ bimester 2019

*n* = *10,780* *1° bimester (Jan-Feb)*	2019	2020	*p*-value	Δ
*N. visits to PED, n (%)*	5097 (47.3)	5683 (52.7)	-	11.5%
*Males, n (%)*	2679 (52.6)	3027 (53.3)	0.46	13.0%
*Age* < *2 years, n (%)*	1486 (29.2)	1903 (33.5)	< 0.0001	28.1%
*Age 2–5 years, n (%)*	2019 (39.6)	1946 (34.2)	< 0.0001	-3.6%
*Age 6–11 years, n (%)*	1172 (23.0)	1462 (25.7)	0.001	24.7%
*Age* > *11, n (%)*	420 (8.2)	372 (6.6)	0.001	-11.4%
**Triage code**				
* Red, n (%)*	11 (0.2)	11 (0.2)	1.00	0.0%
* Yellow, n (%)*	548 (10.8)	518 (9.1)	0.03	-5.5%
* Green, n (%)*	3717 (72.9)	3994 (70.3)	0.003	7.5%
* White, n (%)*	821 (16.1)	1160 (20.4)	< 0.0001	41.3%
* Discharge home, n (%)*	4441 (87.1)	5017 (88.3)	0.06	13.0%
* Hospital admission, n (%)*	656 (12.9)	666 (11.7)	0.06	1.5%
* Death, n (%)*	0 (0.0)	0 (0.0)	-	-
**Reason for PED visit**				
* Acute respiratory disease*	2277 (44.7)	2492 (43.9)	0.40	9.44%
* Gastrointestinal disease*	871 (17.1)	908 (16.0)	0.12	4.3%
* Trauma*	429 (8.4)	599 (10.5)	0.0002	39.6%
* Skin disorders*	399 (7.8)	349 (6.1)	0.0004	-12.5%
* Miscellaneous*	196 (3.9)	260 (4.6)	0.07	32.7%
* Neuropsychiatric disease*	231 (4.5)	247 (4.4)	0.80	6.9%
* Fever*	132 (2.6)	138 (2.4)	0.50	4.6%
* Ocular disease*	97 (1.9)	127 (2.2)	0.27	30.9%
* Endocrine and metabolic disorder*	118 (2.3)	100 (1.8)	0.06	-15.3%
* Pain*	82 (1.6)	124 (2.2)	0.02	51.2%
* Cardiac disease*	108 (2.1)	109 (1.9)	0.45	0.9%
* Acute surgical disease*	69 (1.4)	82 (1.4)	1.00	18.8%
* Osteoarticular disease*	69 (1.4)	106 (1.9)	0.04	53.6%
* Intoxication*	19 (0.4)	42 (0.7)	0.04	121.1%

*PED* Pediatric Emergency Department

**Table 3:** Descriptive analysis of demographic and clinical data: 2^nd^ bimester 2020 compared to 2^nd^ bimester 2019

*n* = *6616*	2019	2020	*p*-value	Δ
***2° bimester (Mar-Apr)***				
* No. visits to PED, n (%)*	5683 (82.0)	1192 (18.0)	-	-78.0%
* Males, n (%)*	2988 (55.1)	647 (54.3)	0.62	-78.4%
* Age* < *2 years, n (%)*	1559 (28.7)	402 (33.7)	0.0006	-74.2%
* Age 2–5 years, n (%)*	2043 (37.7)	367 (30.8)	< 0.0001	-82.0%
* Age 6–11 years, n (%)*	1415 (26.1)	323 (27.1)	0.45	-77.2%
* Age* > *11, n (%)*	407 (7.5)	100 (8.4)	0.29	-75.4%
**Triage code**				
* Red, n (%)*	17 (0.3)	1 (0.1)	0.22	-94.1%
* Yellow, n (%)*	496 (9.1)	101 (8.5)	0.51	-79.6%
* Green, n (%)*	4075 (75.1)	906 (76.0)	0.51	-77.8%
* White, n (%)*	836 (15.4)	184 (15.4)	1.00	-78.0%
* Home discharge, n (%)*	4811 (88.7)	962 (80.7)	< 0.0001	-80.0%
* Hospital admission, n (%)*	613 (11.3%)	230 (19.3%)	< 0.0001	-62.5%
* Death, n (%)*	0 (0.0)	0 (0.0)	-	-
**Reason for PED visit**				
* Acute respiratory disease*	2373 (43.8)	375 (31.5)	< 0.0001	-84.2%
* Gastrointestinal disease*	827 (15.3)	166 (13.9)	0.22	-79.9%
* Trauma*	518 (9.6)	211 (17.7)	< 0.0001	-59.3%
* Skin disorders*	513 (9.5)	57 (4.8)	< 0.0001	-88.9%
* Miscellaneous*	251 (4.6)	100 (8.4)	< 0.0001	-60.2%
* Neuropsychiatric disease*	215 (4.0)	56 (4.7)	0.27	-74.0%
* Fever*	166 (3.1)	38 (3.2)	0.86	-77.1%
* Ocular disease*	75 (1.4)	27 (2.3)	0.02	-64.0%
* Endocrine and metabolic disorder*	101 (1.9)	39 (3.3)	0.003	-61.4%
* Pain*	105 (1.9)	35 (2.9)	0.03	-66.7%
* Cardiac disease*	97 (1.8)	15 (1.3)	0.23	-84.5%
* Acute surgical disease*	68 (1.3)	42 (3.5)	< 0.0001	-38.2%
* Osteoarticular disease*	88 (1.6)	13 (1.1)	0.20	-85.2%
* Intoxication*	27 (0.5)	18 (1.5)	0.0001	-33.3%

*PED* Pediatric Emergency Department

**Table 4:** Descriptive analysis of demographic and clinical data: 3^rd^ bimester 2020 compared to 3^rd^ bimester 2019

*n* = *6695*	2019	2020	*p*-value	Δ
***3° bimester (May-Jun)***				
* No. visits to PED, n (%)*	5171 (77.2)	1524 (22.8)		-70.5%
* Males, n (%)*	2817 (54.5)	807 (53.0)	0.30	-71.4%
* Age* < *2 years, n (%)*	1473 (25.8)	499 (32.7)	< 0.0001	-66.1%
* Age 2–5 years, n (%)*	1921 (37.2)	434 (28.5)	< 0.0001	-77.4%
* Age 6–11 years, n (%)*	1446 (28.0)	478 (31.4)	0.01	-66.9%
* Age* > *11, n (%)*	331 (6.4)	113 (7.4)	0.17	-65.9%
**Triage code**				
* Red, n (%)*	8 (0.2)	8 (0.5)	0.05	0.0%
* Yellow, n (%)*	380 (7.4)	108 (7.1)	0.69	-71.6%
* Green, n (%)*	3854 (74.5)	1159 (76.1)	0.21	-69.9%
* White, n (%)*	929 (18.0)	249 (16.3)	0.13	-73.2%
* Home discharge, n (%)*	4621 (89.4)	1260 (82.7)	< 0.0001	-72.7%
* Hospital admission, n (%)*	550 (10.6)	264 (17.3)	< 0.0001	-52.0%
* Death, n (%)*	0 (0.0)	0 (0.0)	-	-
**Reason for PED visit**				
* Acute respiratory disease*	1798 (34.8)	191 (12.5)	< 0.0001	-89.4%
* Gastrointestinal disease*	900 (17.4)	262 (17.2)	0.86	-70.9%
* Trauma*	682 (13.2)	367 (24.1)	< 0.0001	-46.2%
* Skin disorders*	614 (11.9)	169 (11.1)	0.39	-72.5%
* Miscellaneous*	174 (3.4)	103 (6.7)	< 0.0001	-40.8%
* Neuropsychiatric disease*	204 (4.0)	103 (6.8)	< 0.0001	-49.5%
* Fever*	114 (2.2)	48 (3.2)	0.03	-57.9%
* Ocular disease*	191 (3.7)	67 (4.4)	0.21	-64.9%
* Endocrine and metabolic disorder*	98 (1.9)	33 (2.2)	0.46	-66.3%
* Pain*	99 (1.9)	32 (2.1)	0.62	-67.7%
* Cardiac disease*	90 (1.7)	29 (1.9)	0.60	-67.8%
* Acute surgical disease*	81 (1.6)	61 (4.0)	< 0.0001	-24.7%
* Osteoarticular disease*	94 (1.8)	26 (1.7)	0.80	-72.3%
* Intoxication*	32 (0.6)	33 (2.2)	< 0.0001	3.3%

*PED* Pediatric Emergency Department

**Table 5:** Descriptive analysis of demographic and clinical data: 2^nd^ bimester 2020 compared to 1^st^ bimester 2020

	***1° bimester (Jan-Feb)* 2020**	***2° bimester (Mar-Apr)* 2020**	***p*-value**	**Δ**
*No. visits to PED, n (%)*	5683	1192		-79.0%
*Males, n (%)*	3027 (53.3)	647 (54.3)	0.53	-78.6%
*Age* < *2 years, n (%)*	1903 (33.5)	402 (33.7)	0.89	-78.9%
*Age 2–5 years, n (%)*	1946 (34.2)	367 (30.8)	0.02	-81.1%
*Age 6–11 years, n (%)*	1462 (25.7)	323 (27.1)	0.32	-77.9%
*Age* > *11, n (%)*	372 (6.6)	100 (8.4)	0.03	-73.1%
**Triage code**				
* Red, n (%)*	11 (0.2)	1 (0.1)	0.46	-90.9%
* Yellow, n (%)*	518 (9.1)	101 (8.5)	0.51	-80.5%
* Green, n (%)*	3994 (70.3)	906 (76.0)	0.0001	-77.3%
* White, n (%)*	1160 (20.4)	184 (15.4)	0.0001	-84.1%
* Home discharge, n (%)*	5017 (88.3)	962 (80.7)	< 0.0001	-80.8%
* Hospital admission, n (%)*	666 (11.7)	230 (19.3%)	< 0.0001	-65.5%
* Death, n (%)*	0 (0.0)	0 (0.0)	-	-
**Reason for PED visit**				
* Acute respiratory disease*	2492 (43.9)	375 (31.5)	< 0.0001	-85.0%
* Gastrointestinal disease*	908 (16.0)	166 (13.9)	0.07	-81.7%
* Trauma*	599 (10.5)	211 (17.7)	< 0.0001	-64.8%
* Skin disorders*	349 (6.1)	57 (4.8)	0.08	-83.7%
* Miscellaneous*	260 (4.6)	100 (8.4)	< 0.0001	-61.5%
* Neuropsychiatric disease*	247 (4.4)	56 (4.7)	0.65	-77.3%
* Fever*	138 (2.4)	38 (3.2)	0.11	-72.5%
* Ocular disease*	127 (2.2)	27 (2.3)	0.83	-78.7%
* Endocrine and metabolic disorder*	100 (1.8)	39 (3.3)	0.0009	-61.0%
* Pain*	124 (2.2)	35 (2.9)	0.14	-71.8%
* Cardiac disease*	109 (1.9)	15 (1.3)	0.16	-86.2%
* Acute surgical disease*	82 (1.4)	42 (3.5)	< 0.0001	-48.8%
* Osteoarticular disease*	106 (1.9)	13 (1.1)	0.06	-87.7%
* Intoxication*	42 (0.7)	18 (1.5)	0.006	-57.1%

*PED* Pediatric Emergency Department

There were no significant differences in gender distribution between the first semester of 2020 and the same period of 2019. A higher percentage of visits of children aged < 2 years (33.4% *vs* 28.8%; *p* < 0.0001) and of those aged 6–11 years (26.9% *vs* 25.7%; *p* = 0.04) was observed during the first semester of 2020 (Table 1). Moreover, a lower frequency of visits of children aged 2–5 years (30.8% *vs* 34.2%; *p* = 0.02), and a higher percentage of visits of children aged > 11 years (8.4% *vs* 6.6%; *p* = 0.03) were described during the lockdown period in comparison with the pre-lockdown period (Table 5). A significantly higher percentage of visits of children aged 6–11 years was observed during the pre-lockdown and the immediate post-lockdown periods (Tables 2 and 4).

**Fig. 2:**
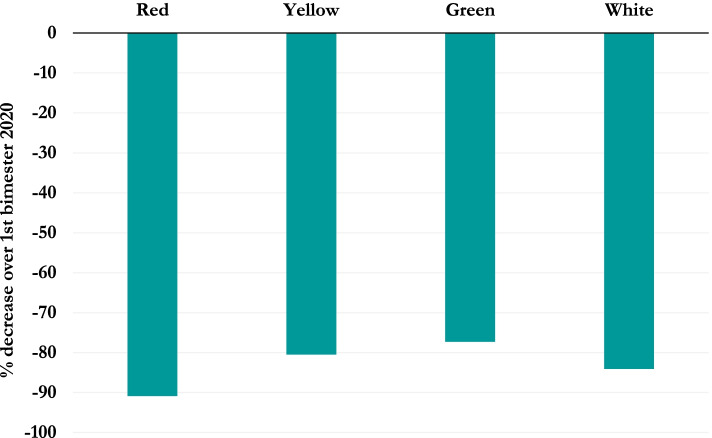
Comparison of triage codes between the first semester of 2020 and the corresponding period of 2019

A lower rate of green (72.1% *vs* 74.2%; *p* = 0.0004) and a higher rate of white codes (19% *vs* 16.5%; *p* < 0.0001) were documented during the first semester of 2020 (Table 1). A higher frequency of white (20.4% *vs* 16.1%; *p* < 0.0001) and a lower of both yellow (9.1% *vs* 10.8%; *p* = 0.03) and green (70.3% *vs* 72.9%; *p* = 0.003) codes were observed during the pre-lockdown months of 2020 when compared with January–February 2019 (Table 2). Moreover, a higher rate of green (76.0% *vs* 70.3%; *p* = 0.0001) and a lower rate of white codes (15.4% *vs* 20.4%; *p* = 0.0001) were observed during the lockdown *vs* the pre-lockdown period (Table 5). The comparisons of triage codes between first and second bimester of 2020 are illustrated in Fig. 2.

The absolute number of hospital admissions was lower (1,160 *vs* 1,819; *p* < 0.0001; Δ:-36.2%), whereas the hospital admission rate was significantly higher (13.8% *vs* 11.6%; *p* < 0.0001) during the 1^st^ semester of 2020 (Table 1).

The increased hospital admission rate was more relevant (19.3% *vs* 11.7%; *p* < 0.0001) when lockdown months were compared with January–February 2020 (Table 5).

A lower proportion of acute respiratory disease (36.4% *vs* 41.1%; *p* < 0.0001) and skin disorder (6.8% *vs* 9.7%; *p* < 0.0001), and a higher proportion of trauma (14% *vs* 10.4%; *p* < 0.0001), acute surgical disease (2.2% *vs* 1.4%; *p* < 0.0001), intoxication (1.1% *vs* 0.5%; *p* < 0.0001), pain (2.3% *vs* 1.8%; *p* = 0.008), neuropsychiatric disease (4.8% *vs* 4.1%; *p* = 0.01) and miscellaneous (5.5% *vs* 4%; *p* < 0.0001) were recorded during the first semester of 2020 (Table 1).

**Fig. 3:**
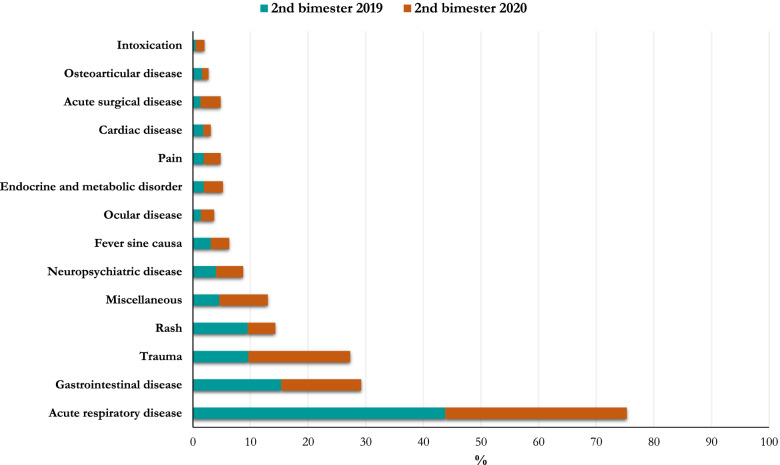
Reasons for pediatric ED visit: second bimester of 2019 *vs* second bimester of 2020

During the lockdown, there was an increased rate of visits for acute surgical disease, trauma, intoxication, endocrine and metabolic disorder, and a significant decrease of visits for acute respiratory disease, as compared to March–April 2019 (Table 3, Fig. 3).

## Discussion

The COVID-19 pandemic have had a significant impact on healthcare systems worldwide.

From March 9^th^ to May 3^rd^, 2020, the Italian Ministry of Health recommended avoiding direct access to the ED in case of fever and/or respiratory symptoms, giving priority to home care or phone consultation for patients with mild or moderate disease [10].

COVID-19 pandemic resulted in a substantial decline of pediatric ED visits and hospitalizations. During March 1–27, 2020, ED visits decreased markedly (∆%: -73 to -88) at 5 Italian pediatric EDs when compared with the same periods in 2019 and 2018 [4]. Similarly to other Authors [7, 11, 12], we observed a marked decrease (∆%: -46.5) in the total number of pediatric ED visits during the first semester of the year 2020, compared to the same period of 2019. The decrease was more relevant when the lockdown period was compared to both the corresponding time period of 2019 (∆%: -78.0), and to the pre-lockdown period (∆%: -79.0).

The lowest number of ED visits in May 2019 might be due to the beginning of the warm season, even though, after May, there is a slight increase in the number of ED visits, consistent with the spread of some viruses (e.g., enteroviruses) in this season. Conversely, in the year 2020, the temporal trend of ED visits showed a marked reduction occurring as early as March–April months, and a slight and gradual increase during May–June. The lowest number of ED visits in April 2020 is consistent with the effect of the lockdown measures, which started one month before.

At the triage assessment, we did not find an increase in urgent triage codes, as previously reported [7, 12, 13]: the percentage of white codes decreased (15.4% *vs* 20.4%; *p* < 0.0001) and, unexpectedly, green codes increased (76% *vs* 70.3%; *p* < 0.0001) during the lockdown months when compared to January–February 2020. Parents seemed to be not discouraged despite national recommendations.

The age of patient ED visits was found to change during the study periods. A higher percentage of visits in children aged < 2 years and a lower in children aged 2–5 years were observed during the first semester of 2020, as proved by other Authors [12, 14]. Unlike the multicentre study by Matera et al. [13], we found no differences for patients older than 11 years. However, unlike other Authors [15], no increased frequency of patients < 2 years was observed after the beginning of the lockdown period.

In line with the study by Vierucci et al. [15], our data showed a progressive decrease of visits for acute respiratory disease from the pre-lockdown period to the post-lockdown period. Moreover, the acute respiratory disease frequency was found to be significantly reduced in both the lockdown and post-lockdown periods when compared to the same periods in 2019, as previously reported [7, 11, 13–17]. In general, the warm season (from June to September) is associated with a decrease of acute respiratory diseases, as found in this study during May–June 2019.

In line with literature, the percentage of children with trauma [7, 12–15], acute surgical problem, intoxication [12, 16], and neuropsychiatric disease [7, 13, 15, 16], was found to be significantly higher in March–April 2020 compared to both March–April 2019 and to January–February 2020.

The lockdown may have contributed to a reduction in community infections, road accidents, and respiratory and cardiovascular diseases due to the interruption of schools and sports activities, the reduction of road traffic and the improvement of air quality [18]. However, social isolation might have exposed children to other risks such as intoxication, neuropsychiatric disorders [11], and trauma [12, 17].

In our study, the hospital admission rate was significantly higher during the lockdown period (with a peak of 19.3%) when compared with that of the same 2019 period; this is consistent with the results of Cozzi G et al. [7], and suggests that children in urgent need of medical care and hospitalization arrived at the pediatric ED despite the lockdown.

Some Authors reported a delayed access to the ED during the lockdown period, resulting in diagnostic and therapeutic delays, and sometimes in fatal outcomes. This could be attributed to the reduced availability of resources [4] and/or to the propensity of caregivers to avoid ED because of fear of infection [4, 19]. The delayed access to healthcare can be especially detrimental to children with special needs, who are at higher risk of severe illness. In the UK and Ireland, Lynn et al. [9] showed that the most common delayed ED presentations were sepsis, diabetes mellitus, and malignancies. On the other hand, the extreme reduction of ED visits (e.g., the inappropriate ones) allowed a better healthcare organization [15].

The main limitations of this study are its retrospective design, the inclusion of only two centres, and the time period considered. The observational, retrospective study design implies that unrecognized factors may have influenced our results. Moreover, the recruited hospitals provide care to a large proportion of pediatric patients in the North and South of Sardinia, but the remaining Sardinian pediatric population was not investigated in this study.

Finally, the division of the observational period into 3 bimesters do not exactly reflect the Italian lockdown period, potentially leading to a mild underestimation of the “lockdown effect”.

## Conclusions

The results of this study seem to be in line with the more recent scientific findings, and show that the SARS-CoV-2 pandemic and associated containment measures have had a profound impact on pediatric ED utilization, with both quantitative (decreased number of visits) and qualitative (variations in the reason for visit and increase in the hospital admission rate) changes.

A better scientific evidence on the multiple effects of SARS-CoV-2 pandemic on pediatric ED visits will be retrieved by large multicentre studies investigating different geographical areas, for longer periods of time.

## Data Availability

The datasets used and/or analyzed during the current study are available from the corresponding author on reasonable request.

## Abbreviations

PED: Pediatric Emergency Department
ED: Emergency Department
SARS-CoV-2: Severe Acute Respiratory Syndrome Coronavirus 2
